# Effects of non-invasive cervical spinal cord neuromodulation by trans-spinal electrical stimulation on cortico-muscular descending patterns in upper extremity of chronic stroke

**DOI:** 10.3389/fbioe.2024.1372158

**Published:** 2024-03-21

**Authors:** Jianing Zhang, Maner Wang, Monzurul Alam, Yong-Ping Zheng, Fuqiang Ye, Xiaoling Hu

**Affiliations:** ^1^ Department of Biomedical Engineering, The Hong Kong Polytechnic University, Kowloon, China; ^2^ Research Institute for Smart Ageing (RISA), Hong Kong SAR, China; ^3^ Research Centre of Data Science and Artificial Intelligence (RC-DSAI), Hong Kong SAR, China; ^4^ Joint Research Centre for Biosensing and Precision Theranostics, Hong Kong SAR, China; ^5^ University Research Facility in Behavioral and Systems Neuroscience (UBSN), The Hong Kong Polytechnic University, Hong Kong SAR, China

**Keywords:** neuromodulation, trans-spinal electrical stimulation, chronic stroke, upper extremity functions, cortico-muscular descending patterns

## Abstract

**Background:** Trans-spinal electrical stimulation (tsES) to the intact spinal cord poststroke may modulate the cortico-muscular control in stroke survivors with diverse lesions in the brain. This work aimed to investigate the immediate effects of tsES on the cortico-muscular descending patterns during voluntary upper extremity (UE) muscle contractions by analyzing cortico-muscular coherence (CMCoh) and electromyography (EMG) in people with chronic stroke.

**Methods:** Twelve chronic stroke participants were recruited to perform wrist-hand extension and flexion tasks at submaximal levels of voluntary contraction for the corresponding agonist flexors and extensors. During the tasks, the tsES was delivered to the cervical spinal cord with rectangular biphasic pulses. Electroencephalography (EEG) data were collected from the sensorimotor cortex, and the EMG data were recorded from both distal and proximal UE muscles. The CMCoh, laterality index (LI) of the peak CMCoh, and EMG activation level parameters under both non-tsES and tsES conditions were compared to evaluate the immediate effects of tsES on the cortico-muscular descending pathway.

**Results:** The CMCoh and LI of peak CMCoh in the agonist distal muscles showed significant increases (*p* < 0.05) during the wrist-hand extension and flexion tasks with the application of tsES. The EMG activation levels of the antagonist distal muscle during wrist-hand extension were significantly decreased (*p* < 0.05) with tsES. Additionally, the proximal UE muscles exhibited significant decreases (*p* < 0.05) in peak CMCoh and EMG activation levels by applying tsES. There was a significant increase (*p* < 0.05) in LI of peak CMCoh of proximal UE muscles during tsES.

**Conclusion:** The cervical spinal cord neuromodulation via tsES enhanced the residual descending excitatory control, activated the local inhibitory circuits within the spinal cord, and reduced the cortical and proximal muscular compensatory effects. These results suggested the potential of tsES as a supplementary input for improving UE motor functions in stroke rehabilitation.

## Introduction

Stroke is the leading cause of permanent motor disabilities in adults, with approximately 75% of individuals experiencing persistent motor deficits in the upper extremity (UE) (Lawrence et al., 2001; Feigin et al., 2022). Poststroke lesions in the brain are diverse among individuals, resulting in different severities of impairment along the corticospinal tract (CST) for motor control (Krakauer and Carmichael, 2022). The impairments weakened or disrupted the coordination of excitatory and inhibitory impulses from the cerebral cortex to the muscles, leading to abnormal descending patterns in the neuromuscular pathways, such as muscle spasticity, muscle weakness, and contralesional compensation at cortical levels (Thibaut et al., 2013; Jones, 2017). Muscle spasticity arises from the impaired inhibitory control in the CST affecting alpha motoneurons in the spinal cord, progressively developed poststroke (Lance, 1980; Kuo and Hu, 2018). As the inhibitory input from the cortex reduces, it causes the hyperexcitability of the alpha motoneurons, usually demonstrated as involuntary muscle contractions in flexors (Kuo and Hu, 2018). Muscle weakness is another common consequence of poststroke CST lesions (Madhavan et al., 2011), related to the alpha motoneurons receive less activation of the descending signals from the lesioned CST, resulting in weakened muscles (commonly extensors) poststroke (Azzollini et al., 2021). In the process of rehabilitation, alternative motor cortical centers could be formed through rehabilitative neuroplasticity in the ipsilesional and/or contralesional hemispheres, introduced mainly by behavioral experiences (Teasell et al., 2005; Dodd et al., 2017). Among the UE muscles, the distal UE (wrist-hand joints) movements are more susceptible to disruption by poststroke spasticity, weakness, and cortical compensatory strategies, as they require a higher degree of precision and control compared to the proximal UE (shoulder-elbow joints) movements (Wissel et al., 2010). Although the objective in the upper limb rehabilitation was to minimize the shoulder/elbow compensations and to improve distal movement, the wrist-hand motor functions often benefited little from the current task-oriented interventions in routine practices, where shoulder/elbow compensations to distal motions were often adopted in a hurry of discharging preparation because of short hospital stays and insufficient professional supervisions for outpatient care (Govender and Kalra, 2007; See; Toh et al., 2022). Furthermore, the descending neural tracts for cortical control to the distal muscles are mainly projected from the ipsilesional hemisphere, with fewer tracts from the contralesional hemisphere than those of the proximal muscles (Ward, 2011). Therefore, it is easy to cause poststroke “learned disuse” in the wrist-hand muscular functions during the progressive neuroplasticity from the central cortex to the muscles, lacking either effective excitatory or inhibitory controls to the distal muscles (Guo et al., 2022).

The residual neural tracts from the ipsilesional hemisphere to the distal wrist-hand muscles could be weak, depending on the severity of the poststroke lesions (Pandian and Arya, 2013). It can be assessed by motor evoked potentials (MEPs) from an agonist muscle by deep brain stimulation (DBS) (Park et al., 2015) and transcranial magnetic stimulation (TMS) (Jo et al., 2016), or cortico-muscular coherence (CMCoh) by electroencephalography (EEG) and electromyography (EMG) during muscular voluntary contractions (Lai et al., 2016). Significantly lower CMCoh intensities and MEP amplitudes, or even the absence of these signals, have been reported in stroke survivors, associated with impairments in the generation of effective neural commands at the cortical level and in the delivery of the residual neural drives in the descending pathways (Classen et al., 1997; Liu et al., 2019). Furthermore, the limited spatial resolution of direct brain stimulation makes it difficult to precisely target the cortical lesion area due to the variability in the location of cortical lesions and the varying degrees of impairments in stroke patients. Currently, there is no immediate method to restore the cortical center in the ipsilesional hemisphere after a stroke. It is mainly because that the Hebbian neuroplasticity enhancement is required by repeated excitation of the focal neurocircuitries through long-term physical training on the one hand (Gharabaghi et al., 2014; Huang et al., 2018) and challenges are faced in direct brain stimulations, such as DBS and TMS, when stroke individuals have heterogeneous cerebral lesions on the other hand (Hara et al., 2021). Compared with direct brain stimulation, targeting the intact cervical spinal cord of stroke patients provides a more straightforward approach to identifying the specific area responsible for controlling upper limb motor function (Pirondini et al., 2022). It has been reported that the delivering efficiency of the residual neural drives to a target distal muscle could be facilitated by modulating the excitatory of the intact spinal cord even when the ipsilesional neural drives are still weak after stroke (Pirondini et al., 2022). Trans-spinal electrical stimulation (tsES) is an emerging technology for non-invasively varying the excitatory threshold of spinal circuitries by delivering electrical current transcutaneously (Gerasimenko et al., 2015; Powell et al., 2023). The rehabilitative effects of tsES were mainly explored in spinal cord injury (SCI) persons but limited in stroke. Attempts have been made in applying tsES to improve neural transmission across the lesional sites after SCI for motor restoration in the upper limbs (Inanici et al., 2021). For example, previous studies have demonstrated that tsES could immediately modulate the excitability of spinal cord circuits and facilitate voluntary control of the triceps muscle in SCI individuals by using rectangular stimulation waveform with 1 ms bursts of 10 kHz carrier frequency at the level of C5-C6 interspinous space (Chandrasekaran et al., 2023). tsES has also been applied to the spinal cord at the C3-C6 spinal segments to assist in fine motor control of upper limbs, i.e., hand pinch and grip strength, in patients with cervical SCI (Inanici et al., 2018). These applications were considered that tsES could provide a sub-threshold activation to the motor neurons in the spinal circuits so that they are closer to the threshold to be easier activated by the residual descending pathways from the cerebral cortex, thus facilitating the impulse propagation through the motor neurons (Minassian et al., 2016; Hofstoetter et al., 2017). Additionally, tsES has decreased muscle spasticity in the upper limb of SCI patients using different stimulation parameters: biphasic rectangular pulses of 30 Hz and stimulation intensity of 20–100 mA for the upper limb (Freyvert et al., 2018). This is mainly related to the direct activation of local inhibitory circuits in the spinal cord via posterior root fibers and the involvement of long-loop processes in descending activation of presynaptic inhibition (spinal-brainstem-spinal mechanisms) (Hofstoetter et al., 2014).

Despite preliminary positive findings have shown tsES could temporarily release spasticity in the upper limb muscles after stroke, the neuromodulatory mechanism to the recovery is unclear (Paget-Blanc et al., 2019). For example, a study explored the effects of direct current stimulation at the C6 spinal segment and observed a potential trend in reducing spasticity of the wrist flexor muscles by suppressing hyperexcitability in the spinal alpha motoneurons after stroke (Paget-Blanc et al., 2019). Another pilot study demonstrated that continuous epidural electrical stimulation to the cervical spine could immediately improve the hand grip force and kinematics on two chronic stroke participants (Powell et al., 2023). Unfortunately, neither of these studies investigated the neuromodulatory effects of tsES on the poststroke neuromuscular systems for the necessary understanding of the potential rehabilitative mechanism related to tsES. Therefore, the objective of this study was to investigate the immediate effects of tsES on the cortico-muscular descending patterns during UE movements on the affected side of individuals with chronic stroke.

## Materials and methods

This study investigated the effects of tsES on cortico-muscular descending patterns in the UE of chronic stroke survivors. Specifically, EEG and EMG measurements were employed to assess the effects of tsES on the affected side during UE motion tasks, i.e., wrist-hand extension and flexion tasks. CMCoh was applied to analyze the coordination patterns between the sensorimotor areas and the upper limb muscles for motor control. The laterality index (LI) of peak CMCoh was used to assess the compensation of the contralesional hemisphere. The EMG activation level was also acquired to evaluate the UE muscle activation patterns.

### Experimental Setup

**FIGURE 1:**
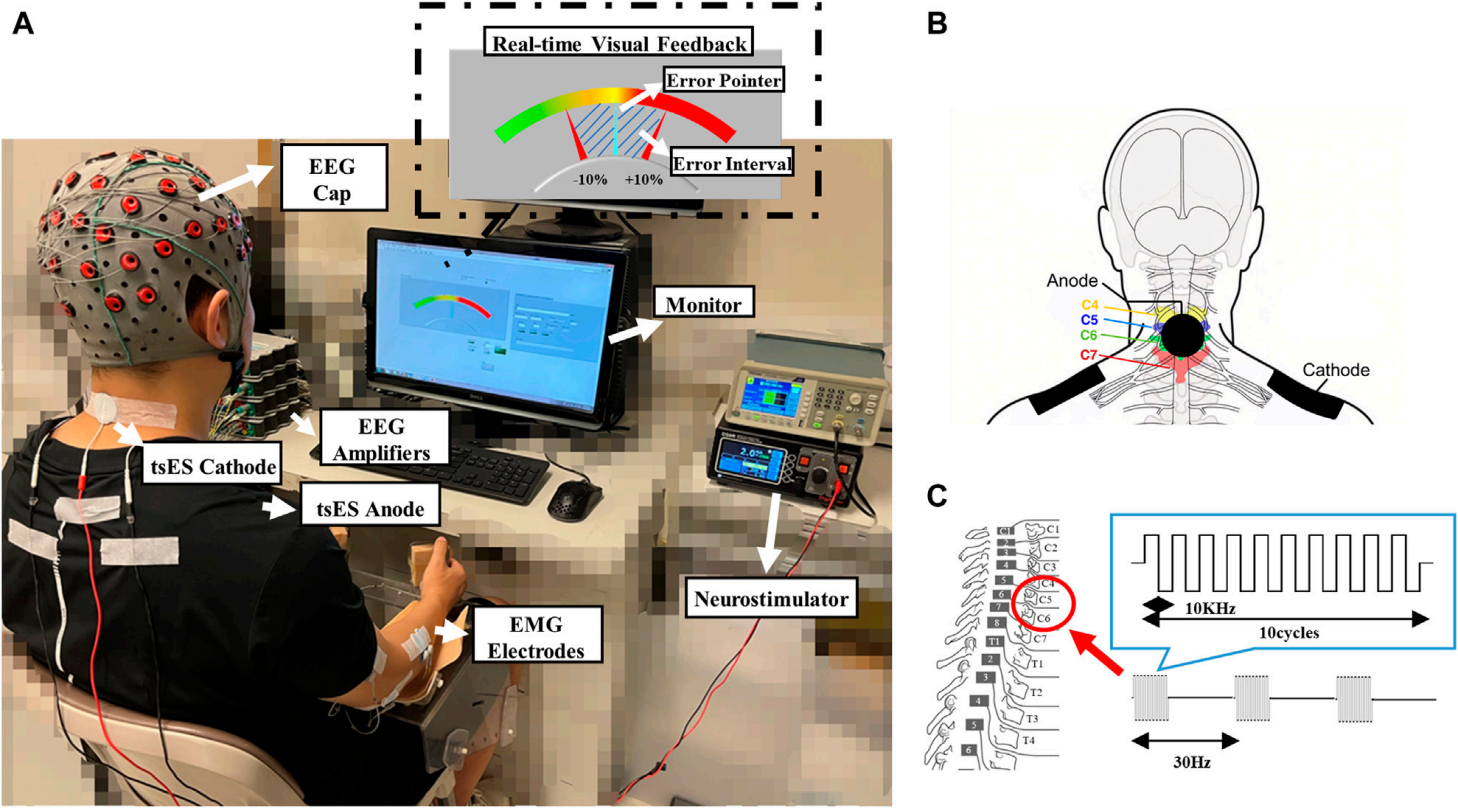
The experimental setup with the tsES. **(A)** A representative stroke participant with the tsES applied to the cervical spinal cord when performing the wrist-hand motion tasks on the affected upper limb; **(B)** Illustration of the attachment of tsES anode and cathode electrodes; **(C)** Illustration of the cervical spinal cord’s electrical stimulation. EEG: electroencephalography; EMG: electromyography; tsES: trans-spinal electrical stimulation.

Figure 1 illustrates the experimental setup, including the configuration of the electrical stimulation site on the cervical spinal cord, the determination of the stimulation intensity, and the attachment of EEG and EMG electrodes. A stroke participant was seated in a comfortable chair facing a 14-inch monitor screen, with the affected UE in a relaxed state (Figure 1A). The forearm of the affected UE was positioned in a neutral orientation on a stable horizontal slab, ensuring that the plane of force exerted by the hand was perpendicular to gravitational force (Johanson et al., 1990). The implementation of tsES on the cervical spinal cord was achieved using a DS8R constant current neurostimulator (Digitimer, Hertfordshire, UK) (McGeady et al., 2022b). As illustrated in Figures 1A,B circular cathode electrode with a diameter of 3 cm (ValuTrode, Axelgaard Manufacturing Co., Ltd., USA) was positioned in the C4-C6 intervertebral space. Two rectangular 8.5 × 6 cm inter-connected anode electrodes (Guangzhou Jetta Electronic Medical Device Manufacturing Co., Ltd., China) were placed bilaterally over the acromioclavicular joints (Barss et al., 2020; McGeady et al., 2022a; McGeady et al., 2022b). The cervical spinal nerves at the C4-C6 intervertebral space were selected for stimulation due to their role in providing motor control and sensation to the UE muscles based on their branching spinal levels (Kayalioglu, 2009). Specifically, the cervical spinal nerves at C4-C5 control proximal UE muscles, while those at C5-C6 control distal UE muscles (Di Lazzaro et al., 1992). After determining the stimulation site, the electrical stimulation was applied in bursts consisting of 10 rectangular biphasic pulses, with each lasting 100 µs. These bursts were delivered at a frequency of 30 Hz and accompanied by a carrier frequency of 10 kHz (as displayed in Figure 1C) (Wecht et al., 2021; McGeady et al., 2022b). The stimulation current in the form of rectangular biphasic pulses helped to prevent net charge injection into the stimulated tissue by maintaining the charge balance, thereby reducing the risk of tissue damage (Harnack et al., 2004; Hofmann et al., 2011). The carrier frequency of 10 kHz was utilized to minimize pain perception during the electrical stimulation. This allowed for the application of higher stimulation current intensity (Barss et al., 2022). The parameters of tsES used in this study have been applied to individuals with SCI and traumatic brain injury (TBI) who have reported acceptable pain levels (Militskova et al., 2020; Inanici et al., 2021).

**FIGURE 2:**
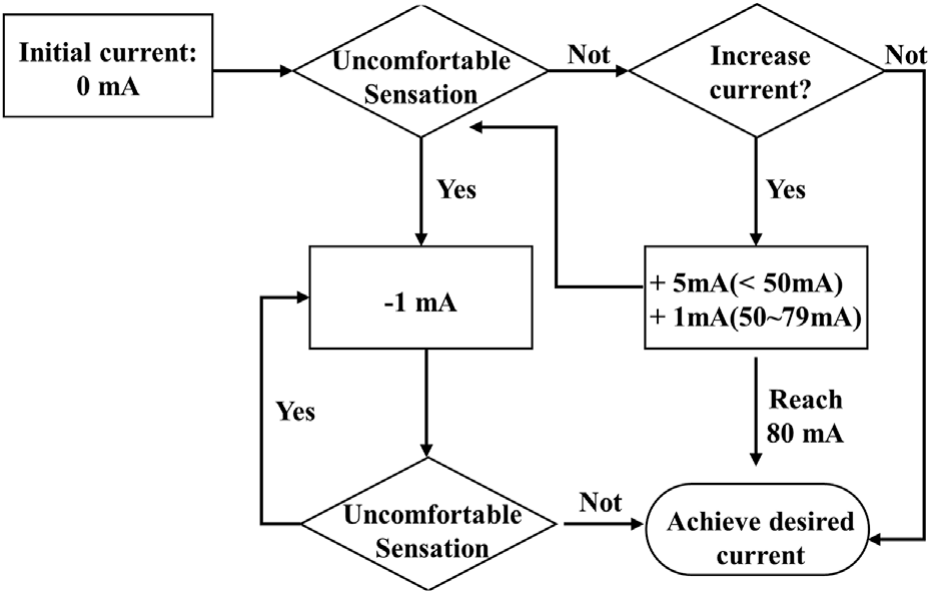
The feedback flowchart for the determination of the stimulation current intensity.

The identification of stimulation intensities for each stroke participant in the study was based on the criteria of maximal tolerable current, as applied in previous studies (Manson et al., 2020; Militskova et al., 2020). Figure 2 illustrates the process of identifying the intensity of stimulation current (in mA) through a feedback loop to determine the maximum tolerance intensity. The procedure began with an initial stimulation current intensity of 0 mA and increased steadily by 5 mA increments from 5 to 50 mA (1 mA increment from 50 to 80 mA for reducing discomfort caused by larger increments) (McGeady et al., 2022b). Before each incremental increase, the stroke participant was asked to confirm his or her ability to tolerate the sensation for at least 30 s. If the intensity was deemed intolerable, it was reduced by one increment and used as the desired stimulation current intensity for motion tasks. To ensure safety and prevent injury, the maximal stimulation intensity threshold for all stroke participants was set at 80 mA and monitored throughout the experiment to ensure that the degree of stimulation supplied to the skin on the cervical spine remained safe according to previous experiments reported on human subjects (Zhang et al., 2020). Additionally, blood pressure and heart rate were monitored every 3 min throughout the determination of the stimulation intensity.

The attachment of EEG electrodes involved mounting a 64-channel EEG cap (g.GAMMAsys, g.tec Gmbh, Austria) on the scalp of a stroke participant. The electrodes were referenced to the left earlobe, and the ground electrode was positioned at AFz (10–20 standard system). The sensorimotor cortex was covered by 21 EEG channels (C1-C6, CZ, CP1-CP6, CPZ, FC1-FC6, FCZ), which were used to record EEG signals. The sensorimotor cortex was selected because it serves as the primary source of the cortico-muscular descending pathway (Zandvoort et al., 2019). EMG signals were collected from five UE muscles. These included three distal muscles: the combined muscle of extensor carpi ulnaris (ECU) and extensor digitorum (ED): ECU-ED; the combined muscle of flexor carpi radialis (FCR) and flexor digitorum (FD): FCR-FD; and abductor pollicis brevis (APB). Additionally, two proximal muscles: biceps brachii (BIC) and triceps brachii (TRI) were also included in EMG signals collection. The EMG signals of each UE muscle were captured using a bipolar configuration with a 2 cm inter-electrode spacing (Ambu, BlueSensor N electrodes, Denmark). The EMG electrodes were referenced to the olecranon of the elbow. Before attaching these EMG electrodes, the skin surface was cleaned using abrasive gel (Bio-Medical Instruments Inc., Warren, United States) to remove the dead skin cells. Following this, the alcohol-soaked cotton pads were used to clean the skin surface and remove oils before electrode placement for both EMG and EEG (Davis, 1959). The electrode-skin impedances of all EMG and EEG channels were maintained below 5 kΩ (Hewson et al., 2003; Xue et al., 2023). The g.USBamp amplifier was used to amplify the EEG signals 10,000 times, and they were then filtered with a bandpass filter that ranged from 2 Hz to 100 Hz (Zhou et al., 2021). The EMG signals were amplified 1000 times using the same amplifier and filtered with a bandpass filter ranging from 10 Hz to 500 Hz (Li et al., 2014). All the EEG and EMG signals were filtered with a 50 Hz notch filter. To capture the synchronized EEG and EMG signals, a USB-6009 DAQ board (National Instruments, Austin, United States) was employed with a sampling frequency of 1,200 Hz.

The online processing during the real-time visual feedback of wrist-hand motion control employed the EMG signals collected from the ECU-ED and FCR-FD. The feedback was provided by a custom operational interface developed using LABVIEW software (National Instruments Corp., United States). As depicted in the upper right corner of Figure 1A, the interface presented a color range from left to right, to visually represent the varying levels of agonist muscle contraction from 0% to 100% isometric maximal voluntary contraction (iMVC) during wrist-hand motion tasks, e.g., ECU-ED for extension, FCR-FD for flexion. The procedure for conducting iMVC measurement was denoted in Section 2.3 Evaluation Protocol. The motion of the blue pointer on the interface corresponded to the immediate fluctuations in the contraction level of the agonist muscles, and the two fixed red pointers indicated the permissible range of ±10% error during motor control (Meng et al., 2008). The agonist muscle i (ECU-ED and FCR-FD) real-time contraction levels were calculated as follows (Guo et al., 2020):

$${\text{EMG}}_{\text{contraction}\left(i\right)}=\frac{{\text{EMG}}_{i}-{\text{EMG}}_{\text{baseline}\left(i\right)}}{{\text{EMG}}_{\max\left(i\right)}-{\text{EMG}}_{\text{baseline}\left(i\right)}}\times 100\%$$ (1)

where EMG_i_ was the average value of the rectified real-time EMG envelope for muscle i in a 100 ms window; EMG_max(i)_ and EMG_baseline(i)_ denoted the average value of the muscle i’s rectified immediate EMG envelope during maximum force and resting state, respectively.

### Subject recruitment

After acquiring the ethical clearance from the Human Subjects Ethics Sub-committee (HSESC) at the Hong Kong Polytechnic University, chronic stroke individuals were recruited with the following inclusion criteria: 1) age ranging from 30 to 70 years; 2) a minimum of 6 months post-unilateral brain lesion caused by stroke; 3) sufficient cognition to comprehend the experiment’s content and basic instructions (Mini-Mental State Examination (MMSE) score >21); 4) moderate muscle tone at the wrist, finger, and elbow (Modified Ashworth Score (MAS) < 3); 5) motor impairments in the UE affected side with Fugl-Meyer Assessment (FMA)-UE score ranging from 15 to 55, with a maximal value of 66, with a maximal value of 66); 6) detectable voluntary EMG signals, indicated by being three times standard deviation above the baseline of the five muscles in the affected unilateral UE; 7) being able to sit up for at least 60 min (with or without assistance). The exclusive criteria were as follows: 1) musculoskeletal dysfunction or tendon surgery in the UE; 2) injection of botulinum toxin in UE muscles within the past 6 months; 3) any metal and electronic implanted stimulator, e.g., cardiac pacemaker, vagus nerve stimulator, cochlear implant, etc.; 4) medications that influence neural excitability, e.g., antidepressants, antiepileptic, antipsychotics, etc.; 5) allergy to the electrode material; 6) epilepsy or pregnancy. Finally, twelve survivors of chronic stroke (Age, 51.7 ± 11.3 years; Stroke onset, 8.8 ± 5.9 years) were recruited from the different districts in Hong Kong through advertisement for this study. All of them gave the written content before the experiment. The demographic details are listed in Table 1.

**TABLE 1:** Demographic characteristics of the chronic stroke subjects.

Subject	Age (years)	Gender (male/female)	Stroke type (H/I)	Affected side (right/left)	Years since stroke	FMA-UE	MAS-wrist
1	63	M	H	Left	6	39	2
2	65	F	I	Right	13	50	1.4
3	51	F	H	Left	8	36	1
4	54	M	H	Left	3	21	3
5	37	M	H	Right	19	45	1.4
6	41	F	H	Right	7	55	1
7	50	F	H	Left	3	49	1
8	59	M	I	Right	11	50	2
9	41	F	H	Right	10	19	2
10	67	M	I	Left	19	35	3
11	34	M	H	Left	3	43	1.4
12	58	F	H	Right	3	43	1.4
Overall	51.7 ± 11.3	6/6	9/3	6/6	8.8 ± 5.9	40.4 ± 11.2	1.7 ± 0.7

**Note:** Data are presented as mean ± SD. H, Hemorrhagic; I: Ischemic. FMA-UE, Fugl-Meyer Assessment on upper extremity; MAS, Modified Ashworth Score.

### Evaluation Protocol

An iMVC measurement was conducted before the sessions to determine the baseline and maximum levels of EMG signal for the visual feedback during the execution of wrist-hand motion tasks for the five muscles. The measurement of agonist muscle in a participant was carried out based on the protocol in (Guo et al., 2020) with a repetition of 3 times: 1) the UE was maintained in a relaxed state for 5 s to record the baseline EMG; 2) the participant was instructed to rapidly produce maximum force of the muscle and maintain the contraction level for 5 s. To prevent muscular fatigue, a 5-min break was allowed between two consecutive contractions. The maximum value of the three iMVC measurements was selected as the maximum EMG level for each UE muscle.

**FIGURE 3:**
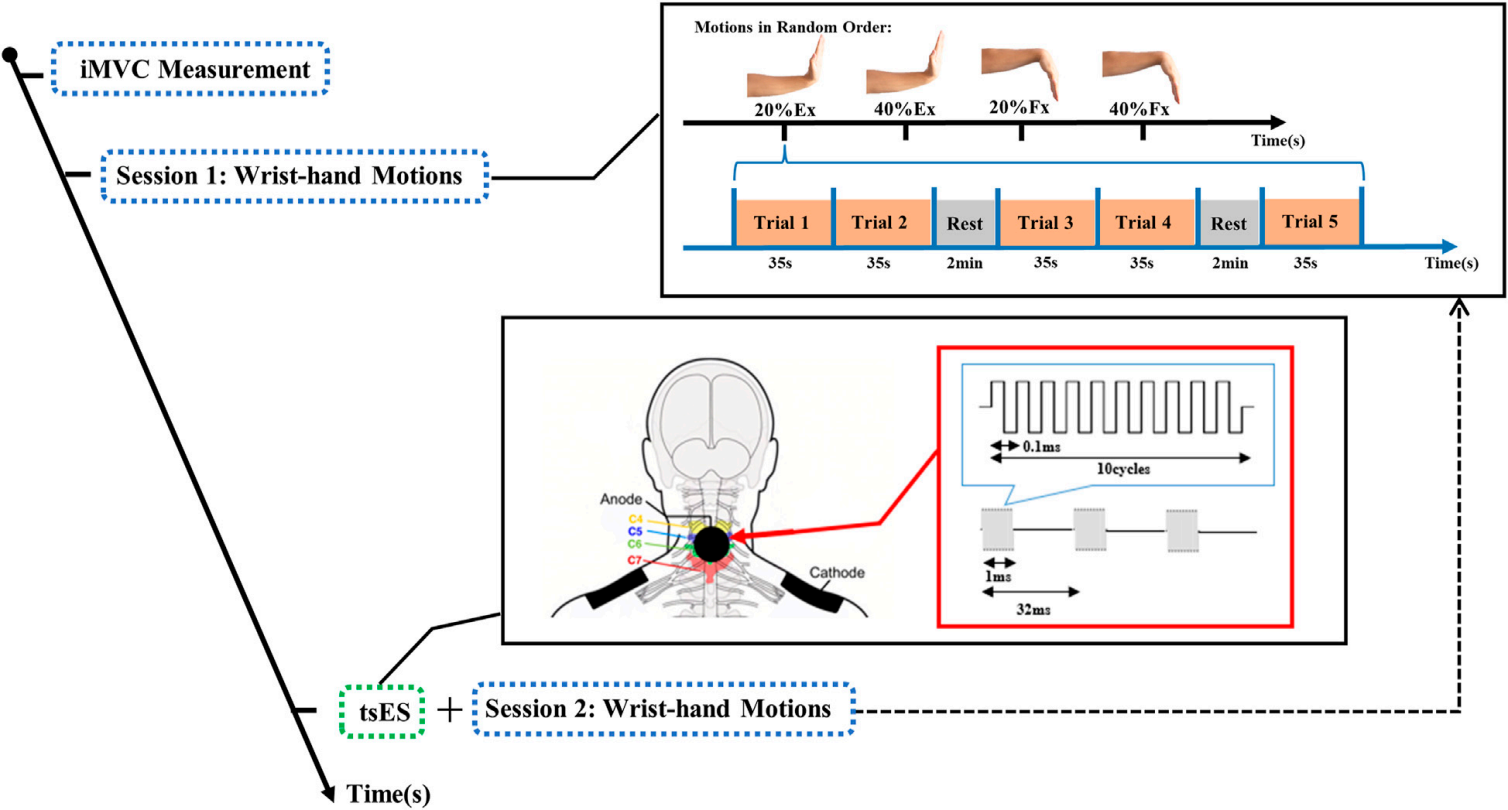
The experimental protocol for the wrist-hand extension and flexion motions with trans-spinal electrical stimulation. Ex: wrist-hand extension; Fx: wrist-hand flexion; iMVC: isometric maximal voluntary contraction; tsES: trans-spinal electrical stimulation.

After the iMVC measurement, two sessions of motion tasks were conducted for the affected side of a stroke participant, as illustrated in Figure 3. The first session involved wrist-hand extension and flexion without tsES. Two different levels of motor contraction were employed, corresponding to 20% and 40% of the individual’s iMVC. The contraction levels below 50% iMVC, e.g., 20% and 40% iMVC selected in the study, are feasible for persons after stroke to achieve in sustained contractions (Zheng et al., 2016). Furthermore, these levels of contraction were found to elicit the most pronounced CMCoh in the beta band (13–30 Hz) (Conway et al., 1995; Kilner et al., 2000). These contraction levels were labeled as 20% Ex, 40% Ex, 20% Fx, and 40% Fx. The stroke participant was instructed to execute wrist-hand contractions according to the names of motion tasks presented on the monitor screen in random order. The optimal motor control was defined as a 0% deviation from the midline for 35 s, with fluctuation maintained within the allowable range of ±10% error. Each motion task was performed for 5 repetitions, and a 2-min rest was incorporated between each repetition to prevent muscle fatigue. The occurrence of muscle fatigue was determined by a 10% reduction in the mean power frequency of the EMG power spectrum (Tecchio et al., 2006; Yamada et al., 2008). No muscle fatigue was observed during the entire wrist-hand motion tasks.

Following the first session of wrist-hand motion tasks, the neurostimulator was turned on by the experimenter. The optimal stimulation current intensity was then determined based on the stroke participant’s feedback according to Section 2.1 Experimental Setup. The average intensity of optimal stimulation current for all the stroke participants was 42.9 ± 13.9 mA, with a range of 12 and 70 mA. Subsequently, the tsES was delivered to the cervical spinal cord, and the stroke participant was instructed to perform the second session of wrist-hand motion tasks, which had the same requirements as the first session. The duration of the tsES application was equal to the duration of the wrist-hand tasks, which was calculated to be 1660 s (4 motion tasks × (5 trials × 35s +2 times of rest × 2 min)). Before each trial in both sessions of wrist-hand motion tasks, the stroke participant was instructed to minimize head movements, eye blinks, and swallowing actions to avoid any motion artifacts in the collection of EEG and EMG signals.

### EEG and EMG processing

**FIGURE 4:**
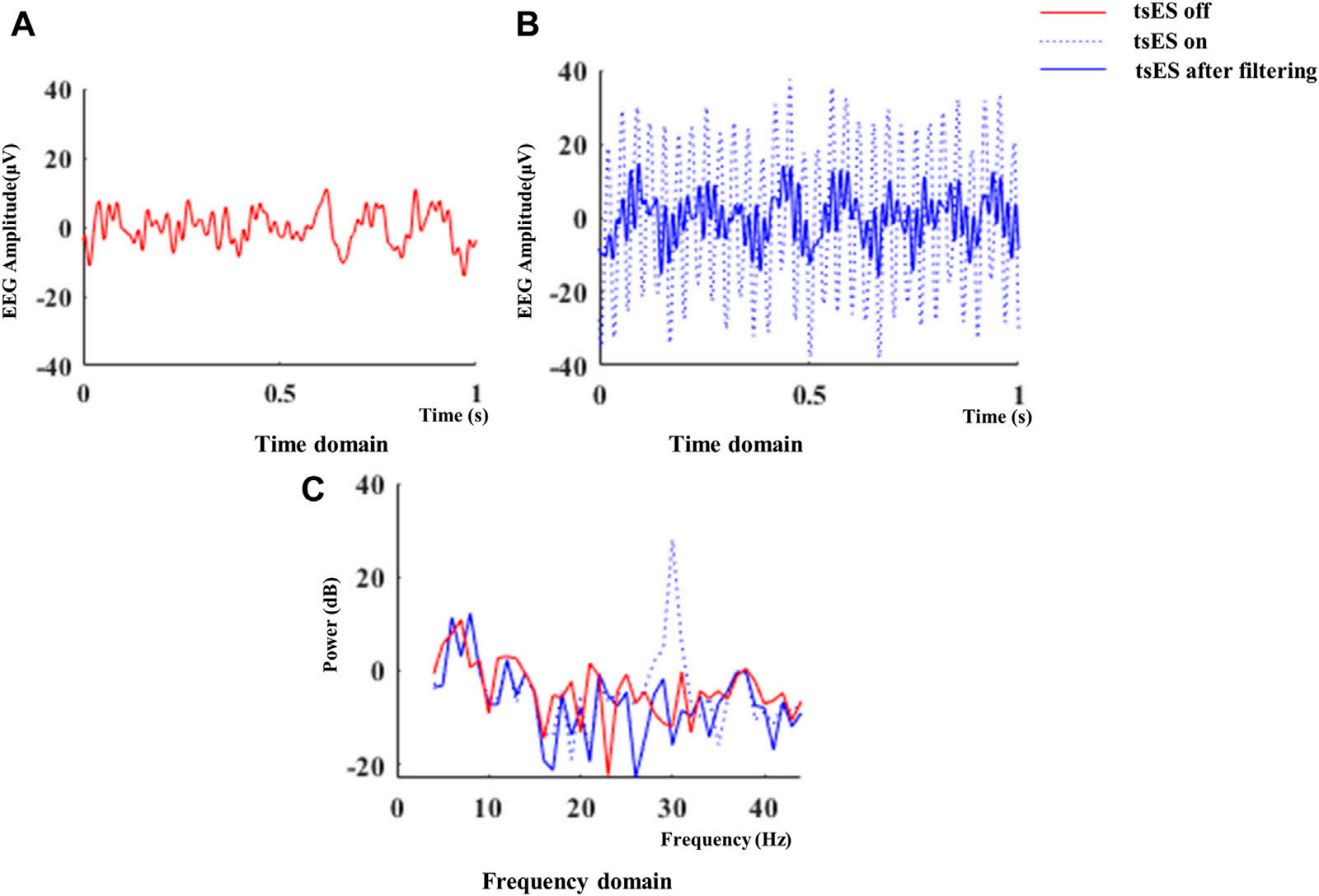
Representative EEG signals recorded in the CZ channel during a 1-s interval of wrist-hand flexion at 20% iMVC level with tsES turned on. The EEG amplitude in the time domain with tsES off and on is shown in **(A)** and **(B)**. The power spectral density of the EEG signals in the frequency domain is also presented in **(C)**. EEG: electroencephalography; tsES: trans-spinal electrical stimulation.

The effects of tsES on cortico-muscular coupling patterns were evaluated by comparing the CMCoh, LI of peak CMCoh, and EMG activation levels under non-tsES and tsES conditions. Independent component analysis (ICA) was applied to remove the eye blink and muscle artifacts from the original EEG signals (Jung et al., 2000). Subsequently, a visual inspection was conducted to ensure that the artifacts in the EEG signals have been adequately removed (Urigüen and Garcia-Zapirain, 2015). The recorded EEG signals were filtered using a third-order Butterworth band-stop filter to eliminate stimulation artifacts during the wrist-hand movement task. Specifically, the band-stop filter from 29 Hz to 31 Hz was adopted to minimize the stimulation artifacts at 30 Hz, as practiced in (McGeady et al., 2021). The electrical stimulation caused a distinct and regular pattern in the recorded EEG signals in the time domain (Figures 4A,B). In the frequency domain, a peak power at 30 Hz was observed in the power spectral density (PSD) of the EEG signals (Figure 4C). After applying the band-stop filter, the EEG spectra appeared similar to those recorded without electrical stimulation, which suggested that the band-stop filter effectively removed the stimulation artifact.

The estimation of cortico-muscular coupling patterns was conducted by analyzing the coherence values between the EEG data of the sensorimotor areas and the EMG data of five UE muscles. CMCoh values at the beta frequency band (13–30 Hz) were calculated as follows:

$${\text{CMCoh}}_{\text{EEG},\text{EMG}}\left(f\right)=\frac{\left|{P_{\text{EEG},\text{EMG}}\left.\left(f\right)\right|}^{2}\right.}{P_{\text{EEG}}\left(f\right)∙P_{\text{EMG}}\left(f\right)}$$ (2)

$$P_{\text{EEG},\text{EMG}}\left(f\right)=\frac{1}{n}\sum_{i=1}^{n}{\text{EEG}}_{i}\left(f\right){\text{EMG}}_{i}^{*}\left(f\right)$$ (3)

where P_EEG,EMG_(f) is the cross-spectrum density of the signals, P_EEG_(f) and P_EMG_(f) are the auto-spectrum densities of the EEG and EMG signals, respectively, at a specific frequency f. The coherence estimation provides a normalized measure of the strength of the cortico-muscular coupling patterns and is expressed as a real number ranging from 0 to 1, where 0 indicates a complete lack of association and 1 indicates a complete correlation between the EEG and EMG signals (Liu et al., 2019). The CMCoh value’s statistical significance (*p* < 0.05) was established if it exceeded the confidence level (CL), which can be calculated using Eq. 4:

$$\text{CL}=1-0.05^{1/\left(L-1\right)}$$ (4)

where L is the number of trial epochs. Each EEG and EMG trial lasted 30 s (originally 35 s, with the final 5 s removed), and was segmented into 1200 data points (1 s) with a 50% overlap. EEG and EMG signals were obtained from 275 trial epochs (55 trial segments × 5 trial numbers). We used a CL of 0.011 (1–0.05^1/(275–1)^ ≈ 0.011) to assess the statistical significance of the CMCoh values. The peak CMCoh values for each UE muscle were measured to identify the most prominent coherence between the EEG and an EMG during UE motion tasks (Omlor et al., 2007). The topography of CMCoh peak values was then used to visualize the cortical activation area with the highest coherence. The locations of CMCoh peak values varied among different stroke participants. The tendency of the CMCoh center towards either the ipsilesional or contralesional hemisphere was quantified by LI, as shown in Eq. 5:

$$\text{Laterality Index}=\frac{{\text{Coh}}_{\text{ipsilesional}}}{\max\left({\text{Coh}}_{\text{contralesional}},{\text{Coh}}_{\text{midsagittal}}\right)}$$ (5)

where Coh_ipsilesional_, Coh_contralesional,_ and Coh_midsagittal_ are the peak CMCoh values in the ipsilesional, contralesional, and midsagittal hemispheres, respectively. LI values less than 1 indicated contralesional hemisphere dominance of peak CMCoh, while LI values greater than 1 indicated ipsilesional hemisphere dominance of peak CMCoh (Park et al., 2011).

The normalized EMG activation levels were utilized to evaluate the patterns of muscle activation during the wrist-hand motion tasks (Chalard et al., 2020). The original EMG signals of muscle i were firstly normalized using the baseline and maximum levels obtained during the iMVC measurement by Eq. 6. The muscle i ’s EMG activation level was then determined using Eq. 7:

$${\text{EMG}}_{\text{Normalized}\left(i\right)}=\frac{{\text{EMG}}_{\text{origin}\left(i\right)}-{\text{EMG}}_{\text{baseline}\left(i\right)}}{{\text{EMG}}_{\max\left(i\right)}-{\text{EMG}}_{\text{baseline}\left(i\right)}}\times 100\%$$ (6)

$${\text{EMG}}_{\text{ActLevel}\left(i\right)}=\frac{1}{T}\int_{0}^{T}{\text{EMG}}_{\text{Normalized}\left(i\right)}\left(t\right)\text{dt}$$ (7)

where EMG_Normalized(i)_ is the normalized EMG of muscle i, $\int_{0}^{T}{\text{EMG}}_{\text{Normalized}\left(i\right)}\left(t\right)\text{dt}$ is the envelope of the EMG of muscle i during over the time interval T, and EMG_ActLevel(i)_ is the muscle i’s EMG activation level after normalization. The EEG and EMG evaluation outcomes were acquired using the custom code with the fieldtrip toolbox (http://www.fieldtrip.fcdonders.nl) based on MATLAB R2019b (The MathWorks Inc., Natick, MA, USA).

### Statistical analysis

The statistical analysis was conducted to evaluate the difference between non-tsES and tsES conditions by using the calculated values of CMCoh, LI of peak CMCoh, and EMG activation levels. These measurements were tested for normality by using the Shapiro-Wilk test. For CMCoh values, both groups exhibited normal distribution at 20% and 40% of Ex and Fx (*p* > 0.05), except for APB at 20% Ex, TRI at 40% Ex, BIC at 20% Fx, and APB at 40% Fx (*p* < 0.05). For the LI of peak CMCoh, both groups showed normal distribution in ECU-ED at 20% Ex and 40% Ex, and FCR-FD at 20% Fx (*p* > 0.05), except for FCR-FD at 40% Fx (*p* < 0.05). For EMG activation levels, both groups had a normal distribution (*p* > 0.05), except for FCR-FD at 20% Ex, BIC at 40% Ex, TRI at 20% Fx and 40% Fx. For the parameters with normal distribution (*p* > 0.05), the paired *t*-test was used to evaluate the differences between tsES and non-tsES conditions. For parameters without normal distribution (*p* < 0.05), the Wilcoxon sign rank test was used to evaluate the differences between tsES and non-tsES conditions. The statistical significance level for this study was defined as 0.05.

## Results

### Cortico-muscular coherence

**FIGURE 5:**
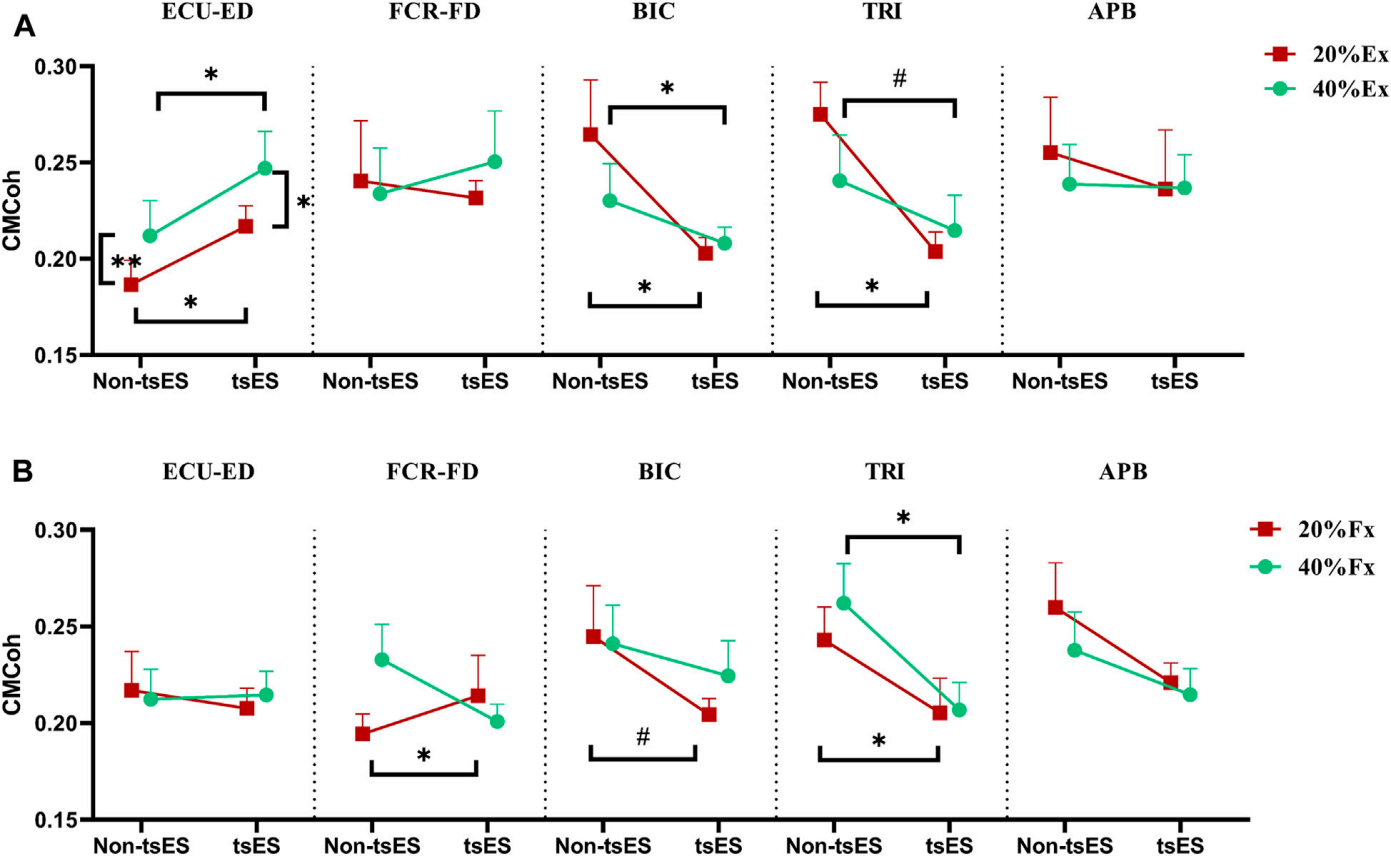
CMCoh at the 20% and 40% contraction levels of iMVC during **(A)** wrist-hand extension and **(B)** wrist-hand flexion under non-tsES and tsES. The CMCoh value is given as mean ± SD. The significant differences are denoted as “*” for *p* < 0.05 (Paired *t*-test), “**” for *p* < 0.01 (Paired *t*-test), and “^***#***^” for *p* < 0.05 (Wilcoxon signed rank test). ECU-ED: the combined muscle of extensor carpi ulnaris (ECU) and extensor digitorum (ED); FCR-FD: the combined muscle of flexor carpi radialis (FCR) and flexor digitorum (FD); BIC: biceps brachii; TRI: triceps brachii; APB: abductor pollicis brevis; CMCoh: cortico-muscular coherence; tsES: trans-spinal electrical stimulation; Ex: wrist-hand extension; Fx: wrist-hand flexion.

Figure 5 displays the CMCoh values of muscles in the UE during both wrist-hand extension and flexion under non-tsES and tsES conditions. Table 2 and Table 3 summarize detailed statistical results of CMCoh values. In the wrist-hand extension, the CMCoh values of ECU-ED significantly increased at both extension contraction levels under tsES (*p* < 0.05, Paired *t*-test). Conversely, the BIC displayed a significant decrease in CMCoh under tsES at both 20% and 40% Ex (*p* < 0.05, Paired *t*-test). Similarly, a significant decrease in CMCoh of TRI was shown at both extension contraction levels (*p* < 0.05, Paired *t*-test and Wilcoxon signed rank test, respectively). In addition, the CMCoh values of ECU-ED significantly increased from 20% Ex to 40% Ex under both tsES and non-tsES. In the wrist-hand flexion, FCR-FD exhibited a significant increase at 20% Fx (*p* < 0.05, Paired *t*-test). In contrast, the TRI and BIC showed significantly decreased CMCoh values at both contraction levels under tsES (*p* < 0.05), except for the CMCoh values of BIC at 40% Fx. However, there were no significant differences in the other inter-group and intra-group comparisons of the CMCoh values of UE muscles.

**TABLE 2:** CMCoh values of UE muscles during wrist-hand extension at the 20% and 40% iMVC contraction levels under non-tsES and tsES.

Muscle		20%Extension	40%Extension	*P* (Cohen’s d)
		CMCoh (Mean ± SD)		
ECU-ED	Non-tsES	0.187 ± 0.009	0.212 ± 0.011	0.009**(0.457)
	tsES	0.217 ± 0.011	0.247 ± 0.019	0.023*(0.618)
	***P*** (Cohen’s d)	0.030*(0.801)	0.036*(0.767)	
FCR-FD	Non-tsES	0.240 ± 0.031	0.233 ± 0.024	0.846 (0.063)
	tsES	0.232 ± 0.020	0.239 ± 0.018	0.321 (0.127)
	***P*** (Cohen’s d)	0.829 (0.070)	0.590 (0.061)	
BIC	Non-tsES	0.265 ± 0.028	0.230 ± 0.019	0.285 (0.340)
	tsES	0.203 ± 0.013	0.208 ± 0.008	0.749 (0.202)
	***P*** (Cohen’s d)	0.048*(0.686)	0.041*(0.397)	
TRI	Non-tsES	0.275 ± 0.037	*0.241 ± 0.024*	*0.878*
	tsES	0.204 ± 0.010	*0.215 ± 0.018*	*0.799*
	***P*** (Cohen’s d)	0.010*(0.579)	**0.047^#^**	
APB	Non-tsES	0.255 ± 0.029	0.239 ± 0.020	0.612 (0.166)
	tsES	*0.236 ± 0.031*	0.237 ± 0.017	*0.610*
	***P*** (Cohen’s d)	*0.241*	0.907 (0.038)	

**Note:** Data are presented as mean ± SD., The significance differences are denoted as “*” for *p* < 0.05 (Paired *t*-test), “**” for *p* < 0.01 (Paired *t*-test), and “#” for *p* < 0.05 (Wilcoxon signed-rank test). The bold values indicate the presence of statistically significant differences in the *p*-values. ECU-ED, the combined muscle of extensor carpi ulnaris (ECU) and extensor digitorum (ED); FCR-FD, the combined muscle of flexor carpi radialis (FCR) and flexor digitorum (FD); BIC, biceps brachii; TRI, triceps brachii; APB, abductor pollicis brevis; CMCoh, cortico-muscular coherence; tsES, trans-spinal electrical stimulation.

**TABLE 3:** CMCoh values of UE muscles during wrist-hand flexion at the 20% and 40% iMVC contraction levels under non-tsES and tsES.

Muscle		20%Flexion	40%Flexion	*P* (Cohen’s d)
		CMCoh (Mean ± SD)		
ECU-ED	Non-tsES	0.217 ± 0.020	0.212 ± 0.015	0.820 (0.074)
	tsES	0.208 ± 0.011	0.215 ± 0.012	0.603 (0.170)
	***P*** (Cohen’s d)	0.717 (0.118)	0.921 (0.032)	
FCR-FD	Non-tsES	0.194 ± 0.010	0.233 ± 0.018	0.376 (0.670)
	tsES	0.214 ± 0.021	0.201 ± 0.009	0.462 (0.155)
	***P*** (Cohen’s d)	0.043*(0.070)	0.206 (0.430)	
BIC	Non-tsES	*0.245 ± 0.026*	0.241 ± 0.019	*0.878*
	tsES	0.204 ± 0.008	0.224 ± 0.018	0.304 (0.345)
	***P*** (Cohen’s d)	***0.045*** ^***#***^	0.538 (0.202)	
TRI	Non-tsES	0.243 ± 0.017	0.262 ± 0.020	0.346 (0.315)
	tsES	0.205 ± 0.017	0.207 ± 0.014	0.869 (0.054)
	***P*** (Cohen’s d)	0.016*(0.477)	0.023*(0.671)	
APB	Non-tsES	0.260 ± 0.023	*0.221 ± 0.018*	*0.445*
	tsES	0.238 ± 0.020	*0.215 ± 0.013*	*0.575*
	***P*** (Cohen’s d)	0.219 (0.417)	*0.445*	

**Note:** Data are presented as mean ± SD., The significance differences are denoted as “*” for *p* < 0.05 (Paired *t*-test), and “#” for *p* < 0.05 (Wilcoxon signed rank test). The bold values indicate the presence of statistically significant differences in the *p*-values. ECU-ED, the combined muscle of extensor carpi ulnaris (ECU) and extensor digitorum (ED); FCR-FD, the combined muscle of flexor carpi radialis (FCR) and flexor digitorum (FD); BIC, biceps brachii; TRI, triceps brachii; APB, abductor pollicis brevis; CMCoh, cortico-muscular coherence; tsES, trans-spinal electrical stimulation.

### Cortico-muscular coherence topography

**FIGURE 6:**
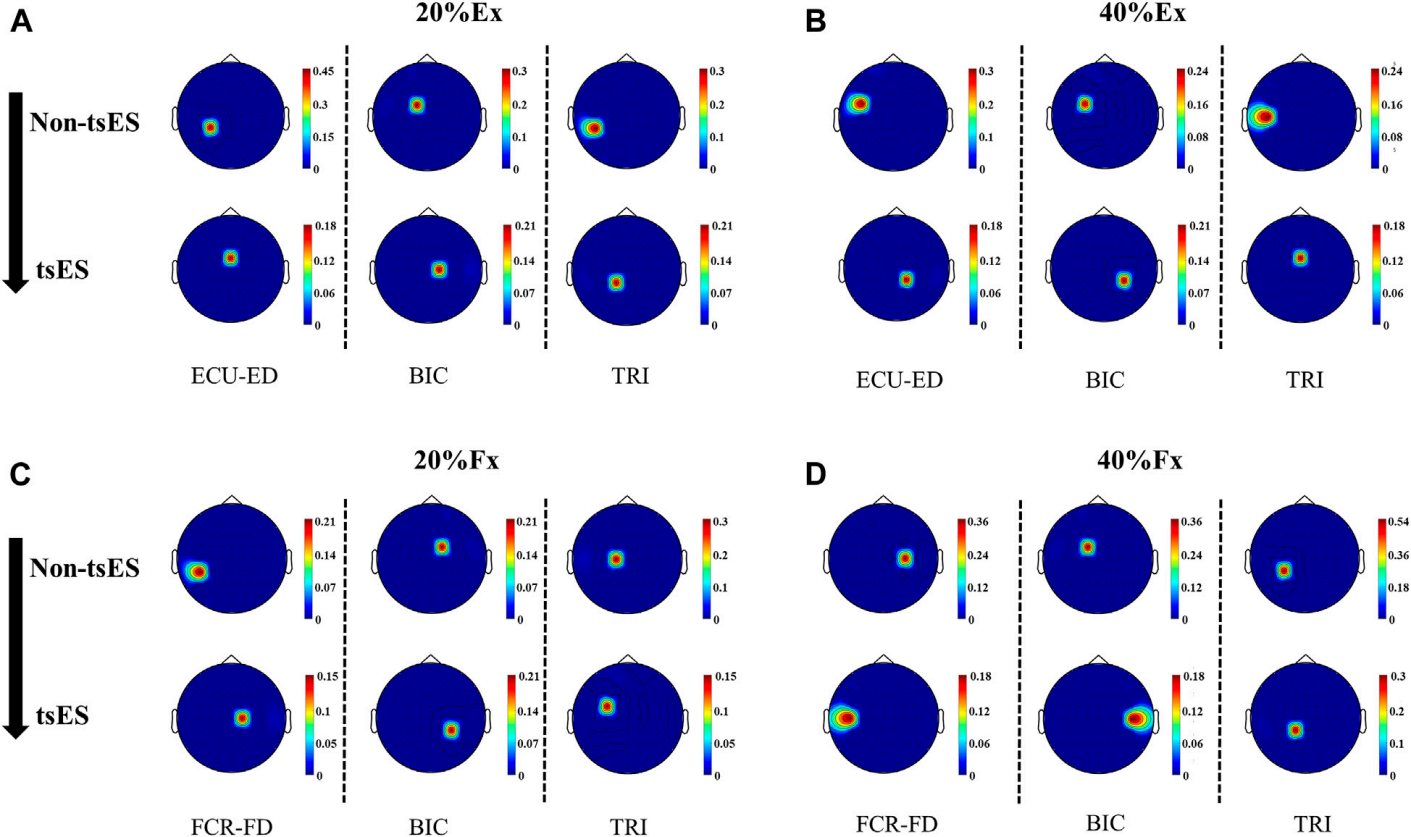
CMCoh topographies of a representative stroke participant with left hemiplegia during the motion tasks, including ECU-ED, BIC, and TRI for **(A)** 20% Ex, **(B)** 40% Ex; FCR-FD, BIC, and TRI for **(C)** 20% Fx, **(D)** 40% Fx under both non-tsES and tsES conditions. ECU-ED: the combined muscle of extensor carpi ulnaris (ECU) and extensor digitorum (ED); FCR-FD: the combined muscle of flexor carpi radialis (FCR) and flexor digitorum (FD); BIC: biceps brachii; TRI: triceps brachii; tsES: trans-spinal electrical stimulation; Ex: wrist-hand extension; Fx: wrist-hand flexion.

Figure 6 presents the peak CMCoh topographies in a representative stroke participant with left hemiplegia. The application of tsES appeared to shift the peak CMCoh channel from the contralesional (left) hemisphere to the ipsilesional (right) sensorimotor cortex in the wrist-hand extension. Specifically, at 20% Ex (Figure 6A), the peak CMCoh channel for ECU-ED, BIC, and TRI shifted from CP3 to FCZ, FC1 to C1, and CP5 to CP1, respectively. At 40% Ex (Figure 6B), the peak CMCoh channel shifted from FC5 to CP2 for ECU-ED, FC3 to CP4 for BIC, and C5 to FCZ for TRI. A similar shift pattern was observed at 20% Fx (Figure 6C) during wrist-hand flexion, where the peak CMCoh channel for FCR-FD and BIC shifted from CP5 to C1, and FC1 to CP4, respectively. At 40% Fx (Figure 6D), the peak CMCoh channel for BIC shifted from FC1 to C5.

**FIGURE 7:**
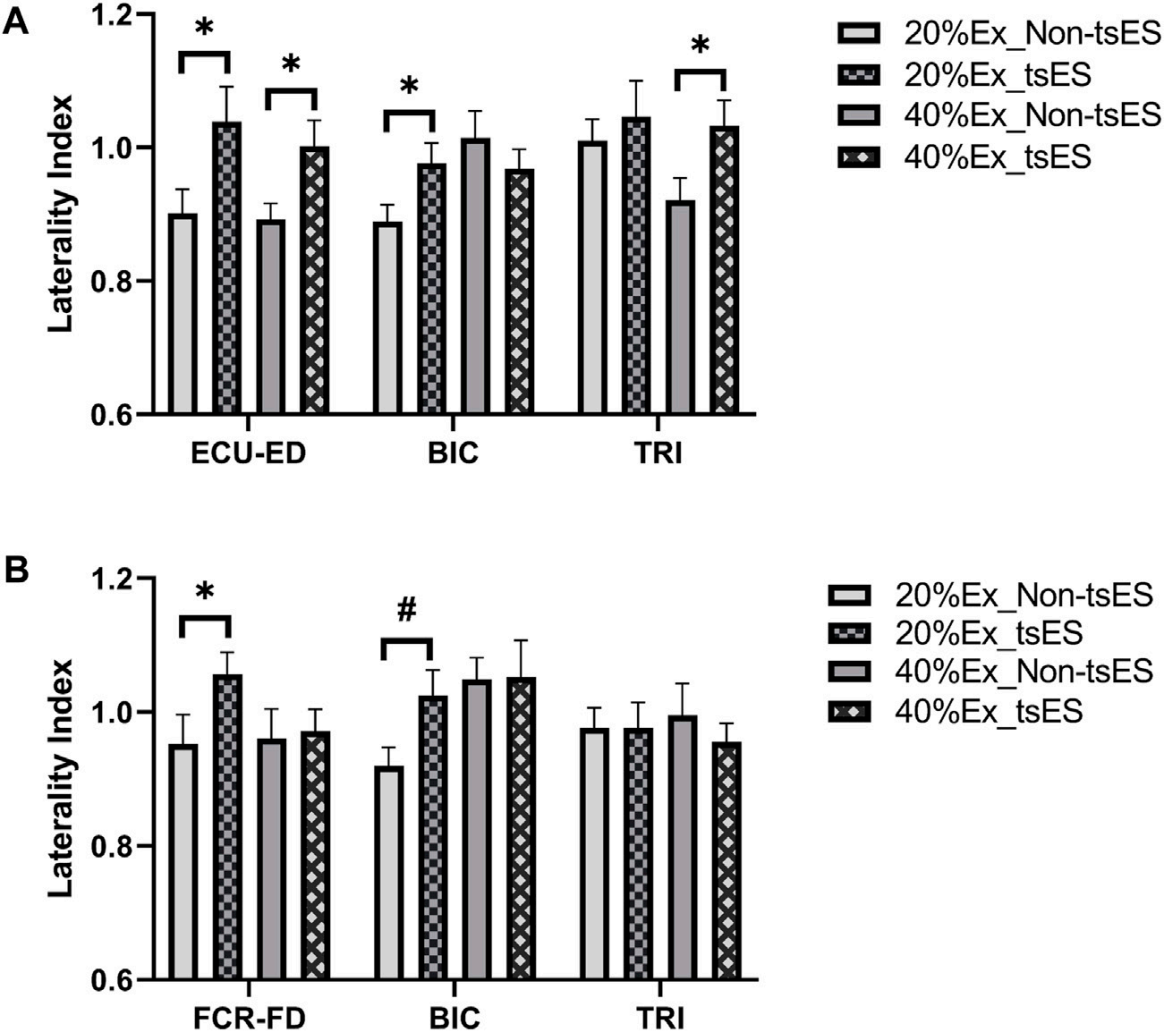
LI of peak CMCoh at the 20% and 40% contraction levels during **(A)** wrist-hand extension and **(B)** wrist-hand flexion under both non-tsES and tsES. LI values are given as mean ± standard deviation. ECU-ED: the combined muscle of extensor carpi ulnaris (ECU) and extensor digitorum (ED); FCR-FD: the combined muscle of flexor carpi radialis (FCR) and flexor digitorum (FD); BIC: biceps brachii; TRI: triceps brachii; tsES: trans-spinal electrical stimulation; Ex: wrist-hand extension; Fx: wrist-hand flexion.

Figure 7 displays the LI values of peak CMCoh on the UE muscles during the wrist-hand motion tasks. Table 4 and Table 5 present detailed statistical information for LI values. Extension motion tasks showed significantly higher LI values in ECU-ED during 20% and 40% Ex, BIC during 20% Ex, and TRI during 40% Ex (*p* < 0.05, Paired *t*-test). Flexion motion tasks also showed significantly higher LI values in FCR-FD (*p* < 0.05, Paired *t*-test) and BIC (*p* < 0.05, Wilcoxon signed rank test) at 20% Fx. There was no significant change in LI in FCR-FD during the 40% Fx.

**TABLE 4:** LI of peak CMCoh during wrist-hand extension at the 20% and 40% iMVC contraction levels under non-tsES and tsES.

Muscle		20%Extension		40%Extension
		Laterality index (Mean ± SD)		
ECU-ED	Non-tsES	0.901 ± 0.036	0.892 ± 0.024	
	tsES	1.039 ± 0.052	1.002 ± 0.039	
	***P*** (Cohen’s d)	0.039*(0.680)	0.040*(0.680)	
BIC	Non-tsES	0.889 ± 0.025	1.015 ± 0.040	
	tsES	0.976 ± 0.031	0.968 ± 0.029	
	***P*** (Cohen’s d)	0.019*(0.796)	0.332 (0.282)	
TRI	Non-tsES	1.010 ± 0.032	0.921 ± 0.033	
	tsES	1.046 ± 0.054	1.033 ± 0.038	
	***P*** (Cohen’s d)	0.325 (0.300)	0.026*(0.742)	

**Note:** Data are presented as mean ± SD., The significance differences are denoted as “*” for *p* < 0.05 (Paired *t*-test). ECU-ED: the combined muscle of extensor carpi ulnaris (ECU) and extensor digitorum (ED); BIC, biceps brachii; TRI, triceps brachii; tsES, trans-spinal electrical stimulation.

**TABLE 5:** LI of peak CMCoh during wrist-hand flexion at the 20% and 40% iMVC contraction levels under non-tsES and tsES.

Muscle		20%Flexion	40%Flexion
		Laterality index (Mean ± SD)	
FCR-FD	Non-tsES	0.952 ± 0.044	0.960 ± 0.045
	tsES	1.056 ± 0.033	0.971 ± 0.033
	***P*** (Cohen’s d)	0.031*(0.711)	0.805 (0.079)
BIC	Non-tsES	0.919 ± 0.028	1.049 ± 0.032
	tsES	1.025 ± 0.038	1.052 ± 0.055
	***P*** (Cohen’s d)	***0.023*** ^**#**^	0.951 (0.014)
TRI	Non-tsES	0.976 ± 0.030	0.995 ± 0.048
	tsES	0.976 ± 0.038	0.955 ± 0.028
	***P*** (Cohen’s d)	0.988 (0.000)	0.446 (0.224)

**Note:** Data are presented as mean ± SD., The significance differences are denoted as “*” for *p* < 0.05 (Paired *t*-test), and “#” for *p* < 0.05 (Wilcoxon signed rank test). The bold values indicate the presence of statistically significant differences in the *p*-values. FCR-FD, the combined muscle of flexor carpi radialis (FCR) and flexor digitorum (FD); BIC, biceps brachii; TRI, triceps brachii; tsES, trans-spinal electrical stimulation.

### EMG activation level

**FIGURE 8:**
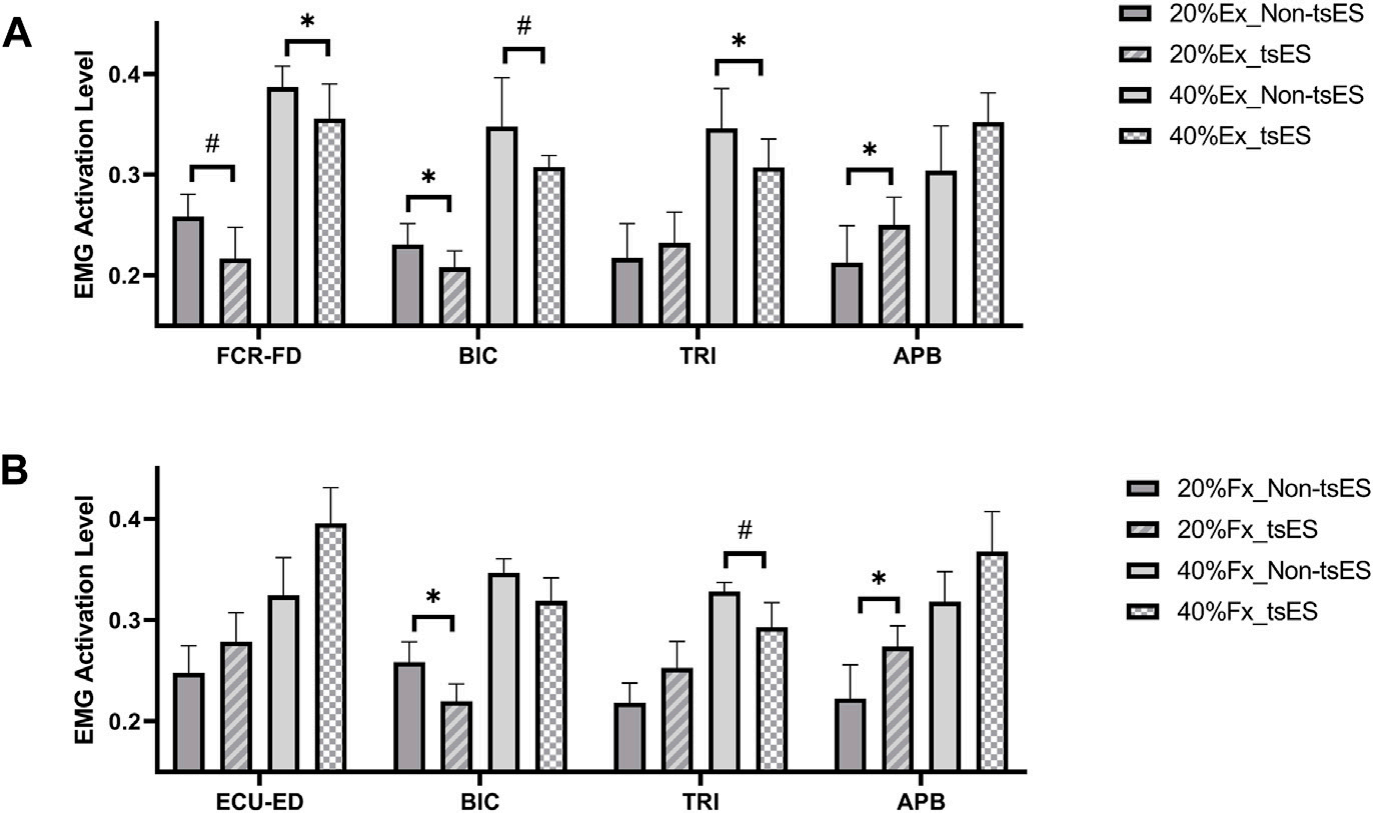
EMG activation levels at the 20% and 40% contraction levels during **(A)** wrist-hand extension and **(B)** wrist-hand flexion under non-tsES and tsES. EMG activation levels are given as mean ± standard deviation. ECU-ED: the combined muscle of extensor carpi ulnaris (ECU) and extensor digitorum (ED); FCR-FD: the combined muscle of flexor carpi radialis (FCR) and flexor digitorum (FD); BIC: biceps brachii; TRI: triceps brachii; APB: abductor pollicis brevis; tsES: trans-spinal electrical stimulation; Ex: wrist-hand extension; Fx: wrist-hand flexion; EMG: electromyography.

Figure 8 presents the EMG activation levels of muscles in the UE during both wrist-hand extension and flexion. The statistical details, e.g., *p*-value and effect size, for EMG activation levels are provided in Table 6 and Table 7. During the 20% Ex, EMG activation levels of FCR-FD (*p* < 0.05, Wilcoxon signed rank test) and BIC (*p* < 0.05, Paired *t*-test) significantly decreased, while the EMG activation level of APB significantly increased (*p* < 0.05, Wilcoxon signed rank test). During the 40% Ex, EMG activation levels were significantly decreased in FCR-FD, TRI (*p* < 0.05, Paired *t*-test), and BIC (*p* < 0.05, Wilcoxon signed rank test). During the 20% Fx, the APB showed a significantly increased EMG activation level (*p* < 0.05, Paired *t*-test). In contrast, the BIC had a significantly decreased trend (*p* < 0.05, Paired *t*-test). During the 40% Fx, the EMG activation level of TRI significantly decreased (*p* < 0.05, Wilcoxon signed rank test). However, no significant differences were found in the other inter-group comparisons of UE muscles’ EMG activation levels.

**TABLE 6:** EMG activation level of UE muscles during wrist-hand extension at the 20% and 40% iMVC contraction levels under non-tsES and tsES.

Motion		FCR-FD	BIC	TRI	APB
		EMG activation level (mean ± SD)			
20%Ex	Non-tsES	0.258 ± 0.022	0.231 ± 0.021	0.217 ± 0.034	0.213 ± 0.037
	tsES	*0.217 ± 0.031*	0.208 ± 0.017	0.232 ± 0.031	0.250 ± 0.027
	***P*** (Cohen’s d)	***0.041*** ^***#***^	0.038*(0.292)	0.542 (0.200)	0.025*(0.385)
40%Ex	Non-tsES	0.387 ± 0.021	*0.348 ± 0.049*	0.346 ± 0.039	0.304 ± 0.044
	tsES	0.356 ± 0.034	0.308 ± 0.011	0.307 ± 0.028	0.352 ± 0.029
	***P*** (Cohen’s d)	0.040*(0.757)	***0.039*** ^***#***^	0.019*(0.363)	0.291 (0.355)

**Note:** Data are presented as mean ± SD., The significance differences are denoted as “*” for *p* < 0.05 (Paired *t*-test), and “#” for *p* < 0.05 (Wilcoxon signed rank test). The bold values indicate the presence of statistically significant differences in the *p*-values. FCR-FD, the combined muscle of flexor carpi radialis (FCR) and flexor digitorum (FD); BIC, biceps brachii; TRI, triceps brachii; APB, abductor pollicis brevis; EMG, electromyography; tsES, trans-spinal electrical stimulation.

**TABLE 7:** EMG activation level of UE muscles during wrist-hand flexion at the 20% and 40% iMVC contraction levels under non-tsES and tsES.

Motion		ECU-ED	BIC	TRI	APB
		EMG activation level (mean ± SD)			
20%Fx	Non-tsES	0.248 ± 0.027	0.259 ± 0.020	0.218 ± 0.020	0.223 ± 0.033
	tsES	0.279 ± 0.011	0.220 ± 0.017	*0.253 ± 0.026*	0.274 ± 0.020
	***P*** (Cohen’s d)	0.405 (0.276)	0.043*(0.744)	*0.445*	0.018*(0.458)
40%Fx	Non-tsES	0.324 ± 0.037	0.347 ± 0.014	*0.328 ± 0.009*	0.318 ± 0.030
	tsES	0.395 ± 0.055	0.319 ± 0.023	0.293 ± 0.023	0.368 ± 0.039
	***P*** (Cohen’s d)	0.067 (0.646)	0.148 (0.501)	***0.033*** ^***#***^	0.368 (0.300)

**Note:** Data are presented as mean ± SD., The significance differences are denoted as “*” for *p* < 0.05 (Paired *t*-test), and “#” for *p* < 0.05 (Wilcoxon signed rank test). The bold values indicate the presence of statistically significant differences in the *p*-values. ECU-ED, the combined muscle of extensor carpi ulnaris (ECU) and extensor digitorum (ED); BIC, biceps brachii; TRI, triceps brachii; APB, abductor pollicis brevis; EMG, electromyography; tsES, trans-spinal electrical stimulation.

## Discussion

This study investigated the immediate effects of tsES on cortico-muscular descending patterns in the UE of chronic stroke individuals during the wrist-hand movement. The differences in CMCoh, LI of peak CMCoh, and EMG activation levels were compared between non-tsES and tsES conditions. The findings revealed that the peak CMCoh shifted towards the ipsilesional hemisphere with tsES. This indicated that tsES could immediately enhance the residual descending pathways originating from the ipsilesional hemisphere and reduce the compensatory effects of the contralesional hemisphere during the motor control of distal UE muscles.

### tsES enhanced excitatory and inhibitory control of the distal UE muscles

Enhanced excitatory descending control of the sensorimotor cortex on the UE muscles was determined by the significantly increased peak CMCoh of agonist muscles, e.g., ECU-ED for 20% Ex and 40% Ex (Figure 5A), FCR-FD for 20% Fx (Figure 5B). These observed changes in CMCoh indicated that tsES had an immediate effect on increasing the control precision of the wrist-hand motion tasks at 20% and 40% iMVC levels of motion difficulties. The underlying mechanism behind this effect involves the modulation of sensory-motor networks within the cervical spinal cord to physiological states that amplify responsiveness to the descending signals from the sensorimotor cortex (Angeli et al., 2014). Previous experimental studies on humans have applied post-activation depression (PAD) to explore the influence of group Ia and group Ib fibers recruitment on the evoked motor potentials of the cervical spinal cord (Milosevic et al., 2019; de Freitas et al., 2022). Their findings confirmed that continuous electrical stimulation preferentially activates and recruits the large-to-medium diameter proprioceptive sensory fibers located in the dorsal root and dorsal column (Willis Jr and Coggeshall, 2012). Compared to alpha motor fibers among neural structures within the vertebral canal, these myelinated axons have lower excitation thresholds, rendering them highly responsive to externally applied electrical stimulation (Rattay, 1999). The activation of these fibers, whose cell bodies are located in the dorsal root of the spinal cord, could convey excitatory potentials to relevant spinal motoneurons and interneurons via monosynaptic and polysynaptic proprioceptive circuits (Koch et al., 2018). Consequently, spinal neurons’ membrane potentials were elevated, which enhanced the responsiveness of spinal circuits to descending signals originating from the sensorimotor cortex (Hofstoetter et al., 2018).

Although the peak CMCoh of antagonist muscles, e.g., FCR-FD for 20% Ex and 40% Ex (Figure 5A), ECU-ED for 20% Fx and 40% Fx (Figure 5B), showed no difference between the non-tsES and tsES conditions for the stroke participants, the EMG activation levels of FCR-FD at both 20% Ex and 40% Ex were significantly decreased with tsES ( Figure 8A ). This reduction in muscular output of the antagonist muscle during wrist-hand extension suggested that the potential activation of local inhibitory circuits within the spinal cord and achieved specifically reciprocal inhibition (Minassian et al., 2007). The reciprocal inhibition is mainly mediated by group Ia afferents, which typically transmit inhibitory signals to the antagonist muscle to suppress its activity during movement (Tanaka, 1974). However, there is evidence of reduced transmission in the reciprocal inhibition pathway in stroke individuals, leading to the alpha motor neurons that control the antagonist muscle being more excitable (Crone et al., 2000; Crone et al., 2003). The electrical stimulation facilitates the depolarization of group Ia afferents in the dorsal roots, which then form strong synaptic connections with inhibitory interneurons in the spinal cord (Jankowska, 1992). These activated inhibitory interneurons could enhance the inhibitory control over the antagonist muscle, reducing its excessive activation and improving the coordination between agonist and antagonist muscles (Hofstötter, 2009). For the CMCoh values that exhibited no significant differences between the non-tsES and tsES conditions, e.g., FCR-FD and BIC for 40% Fx (Figure 5B), APB for all motions (Figures 5A,B), it suggested that either the connection between cortical center and corresponding muscles was weak, or the cortical center did not shift towards to ipsilesional hemisphere with tsES.

### tsES decreased cortical and proximal muscular compensatory effects

The UE proximal muscles, i.e., BIC and TRI, showed a significant decrease in peak CMCoh during wrist-hand extension (Figure 5A) and flexion movements ( Figure 5B) with the application of tsES. It represented relatively reduced cortical resources dedicated to innervating the proximal UE muscles in the distal UE muscle movement. The EMG activation levels of proximal muscles also displayed a decreasing trend with tsES (Figure 8). It demonstrated that the less proximal muscular compensation was involved in the distal UE movements. This could be attributed to the enhanced physiological state of spinal circuits from continuous spinal cord stimulation. This elevation in the physiological state makes the spinal circuits more responsive to the supraspinal commands via the residual descending pathways (Harkema et al., 2011; Hofstoetter et al., 2013). It was further demonstrated by the significantly increased LI in the UE proximal muscles during the wrist-hand motions, i.e., BIC for 20% Ex (Figure 7A) and 20% Fx (Figure 7B), TRI for 40% Ex with tsES (Figure 7A). The shift in hemispheric lateralization towards the ipsilesional hemisphere (Figures 6A–D) reduced the innervation from the contralesional hemisphere to the proximal muscles, thereby diminishing the proximal muscular compensatory effects. Previous studies have investigated the compensatory contractions of proximal UE muscles in the distal UE movements following stroke and have revealed that the cortical control center for proximal UE muscles relocated to the contralesional hemisphere (Guo et al., 2020). Additionally, a functional MRI study has reported increased blood flow in several cortices within the contralesional hemisphere during gripping tasks performed by stroke individuals, indicating a greater degree of contralesional cortex activation compared to unimpaired control participants (Ward et al., 2003). Although proximal-to-distal UE compensation provided a substitution or circumvention for the impaired distal movement, it may exacerbate the ‘learned disuse’, which in turn introduces other motor deficits after stroke, e.g., loss of dexterity and aberrant muscle synergies (Jones, 2017). The poststroke lesions in the primary motor cortex and its descending neural tract leads to a greater impairment in motor control of distal UE muscles, compared to proximal UE muscles. This difference was because the distal UE muscles receive innervation primarily from the lateral corticospinal tract, which originates predominantly from the ipsilesional hemisphere and is more vulnerable to stroke-related lesions. In contrast, the anterior corticospinal tract that controls the proximal UE muscles remains ipsilateral within the spinal cord and is relatively less affected by stroke-related lesions (Zaaimi et al., 2012). The damage to the descending neural tracts is usually partial in most stroke patients and some residual circuits are spared, despite these circuits cannot transmit sufficient excitability to excite the motor neurons in the periphery muscles (Lu et al., 2016; Azzollini et al., 2021). By modulating the spinal cord excitability through electrical stimulation, tsES effectively reduced the threshold for interneurons and motor neurons involved in transmitting motor impulses to facilitate the integration of the remaining descending drive (Pirondini et al., 2022). This integration allows for more propagation of motor control signals from the remaining descending pathways (D’amico et al., 2014). Therefore, the cortical compensation from the contralesional hemisphere to innervate the proximal muscles during the distal movement was decreased.

The observed increased LI in the UE distal muscles during the wrist-hand motions, i.e., ECU-ED for 20% Ex and 40% Ex (Figure 7A), FCR-FD for 20% Fx (Figure 7B) provided further evidence of the enhanced residual descending control from the ipsilesional hemisphere (Figures 6A–D). As previously discussed, the current stimulation of the spinal cord could modulate the functional state of the spinal circuits, facilitating interactions between the descending motor signals of the ipsilesional areas and the innervated distal UE muscles (Zaaimi et al., 2012). This was consistent with previous findings that the strength and control of hand were elevated because of continuous stimulation of the cervical spinal cord (Gad et al., 2018). Specifically, the SCI individuals were able to generate greater hand grip force, and the spinal cord evoked response in distal UE muscles increased while activation in proximal UE muscles decreased by using a multi-segmental stimulation at C3-C4 and C6-C7 levels (Gad et al., 2018). The improvement in the UE muscle synergies during the distal movements may be attributed to the enhancement of synaptic plasticity from the ipsilesional hemisphere (Hamid and Hayek, 2008). Synaptic plasticity involves the connectivity among the descending motor axons, motor neurons, and interneurons in the spinal cord (Hamid and Hayek, 2008). There is a process of synaptic plasticity strengthening when the brain’s descending impulses reach the corticospinal anterior horn, which has been in an excitability state elicited by the electrical stimulation (Rushton, 2003). As a result, this process leads to an increased probability of subsequent neuronal firing following the principle of the Hebbian-type learning effect, wherein repetitive stimulation induces an enhancement of synaptic efficiency (Rushton, 2003).

One potential limitation of this study is the relatively small sample size. The recruitment of participants continued until significant differences between the tsES and non-tsES conditions were achieved in majority of the evaluation parameters. In this work, 12 participants with diverse motor impairments were finally recruited, and the findings indicated that tsES effectively modulated poststroke neuromuscular systems. The neuroimaging data on the precise lesional locations of the participants are not accessible in the study. Their motor impairments were evaluated based on the behavioral assessments, e.g., FMA, on the hemiplegic side. Although the recruited participants showed a wide range in the motor impairments, statistically significant findings were obtained from the 12 recruited participants, when comparing the tsES and non-tsES conditions. It suggested that tsES could be an effective neuromodulator for persons with varied impairments poststroke. In future studies, a larger sample of participants will be recruited and the modulatory effects of tsES on different subtypes of chronic stroke will be investigated by categorizing the participants based on their lesion locations and impairment levels for further understanding on the differences among subgroups. Clinical trials will be conducted to investigate the rehabilitative effects of cervical tsES on the affected upper limb in chronic stroke individuals through multi-session training that combines tsES with voluntary physical training.

In the present study, we investigated the immediate effects of tsES on cortico-muscular descending patterns during dynamic muscular contractions of the affected distal UE in chronic stroke survivors with different stroke types and impairment levels by using electrophysiological measurements, including CMCoh, LI of peak CMCoh, and EMG activation levels. The results demonstrated that the continuous electrical stimulation to the cervical spinal cord could enhance both excitatory and inhibitory control of the UE muscles while reducing the cortical and proximal muscular compensatory effects. Specifically, by modulating the sensory-motor networks in the cervical spine, tsES enhanced the descending excitatory control to the agonist muscle and local inhibitory control to the antagonist muscle. The observed shift in hemispheric lateralization towards the ipsilesional hemisphere and decreased EMG activation levels of proximal UE muscles indicated a reduction in cortical and proximal muscular compensation during distal UE movements. These findings suggested that tsES could immediately enhance responsiveness to the descending signals from the ipsilesional cortex by modulating cervical spinal cord excitability, highlighting its potential as a supplementary input for improving UE motor functions in stroke rehabilitation.

## Data Availability

The original contributions presented in the study are included in the article/Supplementary material, further inquiries can be directed to the corresponding author.
